# Unsedated Endoscopy: Is it Feasible?

**DOI:** 10.4103/1319-3767.70605

**Published:** 2010-10

**Authors:** Abdulrahman M. Aljebreen

**Affiliations:** Gastroenterology Division, Internal Medicine Department, King Khalid University Hospital, King Saud University, Riyadh, Saudi Arabia

Diagnostic gastroscopies are the most commonly performed endoscopic procedures with an incidence of about 8.6 per 1000 of population.[1] The use of conscious sedation has resulted in the widespread acceptance of this procedure among both physicians and patients; however, these sedatives frequently cause significant oxygen desaturation, occasionally a cardiopulmonary complication and rarely death. Arrowsmith et al. reported that 1 in 200 American patients undergoing endoscopy experience a cardiorespiratory complication as a direct result of sedation.[2]

Sedation is estimated to be directly responsible for between 30 and 50% of all equipment, supply and labor costs associated with diagnostic upper gastrointestinal endoscopy.[3]

Intravenous sedation usage varies widely between different countries and cultures. Sedation is rarely used in Japan or other Asian countries, the Middle East and South America. Unsedated endoscopy is also the norm in most European countries including Germany, Greece, Spain, Sweden and Switzerland.[4] In contrast, up to 98% of the American patients undergoing gastroscopy receive sedation.[5]

In a British study, the sedation rate for out-patient diagnostic endoscopy decreased by 54%, from as high as 70% in 1990 to 32% in 1998 (*P* < 0.0001).[6]

In general, there is evidence that the low prevalence of unsedated endoscopy is due more to patient reluctance rather than physician preference.[7]

A double-blind Finnish study compared intravenous midazolam alone with each of three other groups: a placebo-controlled no sedation group, a placebo-controlled pharyngeal local anesthetic group and a third control group that was unblended.[8] The patients in the midazolam group were found to be more likely not to remember the procedure and reported more willingness to return for a repeat procedure. The effects were most pronounced in younger patients. In terms of endoscopist assessment, the patients in the midazolam group were rated as easier to intubate by the endoscopist compared with those in the placebo group, but there was no difference between the midazolam group and either the pharyngeal anesthesia or control groups. Interestingly, the midazolam group had a higher endoscopist rating for overall difficulty and retching during the procedure compared with the pharyngeal anesthesia group.

Another study showed that performing endoscopic ultrasound without sedation, even though was less well tolerated, did not lead to longer procedure times, higher risks or increased reluctance to undergo a repeat procedure.[9]

In this issue of this journal, Sachdeva et al. have shown in a prospective, single-blinded study that although the endoscopist felt more comfortable with sedated versus unsedated gastroscopies, there was no significant difference between the two groups in terms of procedural ease or patient satisfaction.[10]

There are many reasons why some patients prefer to undergo gastroscopy without sedation. In our experience, the most common reasons for the patients not opting for unsedated gastroscopy are the lack of requirement for an escort requirement, the fear of the usual sedation-related complications and restrictions on activities for almost one full day. There is another group of patients who want to know the result of their gastroscopy on the spot and who do not want to feel anxious waiting for their next visit. Contrary to the belief of many endoscopists, the time to complete the gastroscopy is comparable in sedated and unsedated gastroscopy. There is, however, a huge difference in the total time from admission to the endoscopy room to eventual discharge (96 and 6 minutes, respectively; our unpublished data).

Finally, we believe, when enough time is taken to address all of these differences with the patients, many patients would consider unsedated gastroscopy.
